# Significant associations between high-risk sexual behaviors and enterotypes of gut microbiome in HIV-negative men who have sex with men

**DOI:** 10.1128/msphere.00232-25

**Published:** 2025-06-25

**Authors:** Kangjie Li, Xinjing Liu, Xiaohua Zhong, Haijiao Zeng, Tian Liu, Bing Lin, Pinyi Chen, Biao Xie, Xiaoni Zhong

**Affiliations:** 1 School of Public Health, Research Center for Medicine and Social Development, Chongqing Medical University 12550, Chongqing, China; 2 Hospital of Sichuan International Studies University, Chongqing, China; 3 Chongqing Medical University 12550, Chongqing, Chongqing, China; National Institute of Advanced Industrial Science and Technology, Tsukuba, Ibaraki, Japan

**Keywords:** MSM, gut microbiome, enterotype, random forest machine learning

## Abstract

Gut microbiome of men who have sex with men (MSM) exhibits distinctive characteristics compared with general populations. The dysbiosis of the gut microbiome in MSM is also associated with the onset and evolution of HIV infection. Enterotype is an important feature of the gut microbiome and remains unaffected by demographic factors. However, the enterotypes of gut microbiome in MSM are unclear. The associations between enterotypes and high-risk sexual behaviors in this population also remain to be elucidated. HIV-negative MSM were recruited in this study. Fecal samples of the participants were collected and subjected to 16S rRNA gene sequencing. Enterotype clusters were determined by Jensen-Shannon divergence based on genus-level relative abundance. Microbial function predictions were conducted by PICRUSt2 software. Univariate and multivariate logistic regression approaches were utilized to analyze the associations of enterotypes with sexual behaviors. A three-category random forest machine learning model was performed to further examine the correlation between abundant microbiome in each enterotype cluster and anal sex roles. Two enterotype clusters were identified in our data sets, primarily driven by genera *Phocaeicola* and *Segatella*. The alpha diversity was comparable between the two enterotype clusters. Microbial metabolic functions significantly differed, and multivariate logistic regression indicated a significant association between anal sex role and enterotype. The results of the three-category random forest model indicate that the dominant bacterial communities in gut enterotypes can effectively differentiate MSM who engage exclusively in receptive anal intercourse from those who engage in insertive or versatile anal intercourse (AUC: 0.6400, 0.6929, respectively). We identified two enterotype clusters of gut microbiome in HIV-negative MSM. Enterotypes of MSM were significantly associated with anal sex roles. These findings further highlight the close correlation between the gut microbiome and anal intercourse roles.

**IMPORTANCE:**

Our study’s discovery that gut microbiome enterotypes are significantly associated with anal sex roles in HIV-negative MSM opens a new frontier in understanding the complex interplay between microbiology and sexual health. This finding underscores the urgency of delving into the mechanistic connections between the gut microbiome, sexual behaviors, and HIV infection. By identifying modifiable factors influencing gut microbiome composition, we have paved the way for developing personalized preventive strategies that could disrupt the transmission dynamics of HIV within this high-risk population. This research contributes to the fundamental understanding of the gut microbiome’s role in the sexual health of MSM, making it a pivotal advancement in the fields of gut microbiome research and sexual health.

## INTRODUCTION

Men who have sex with men (MSM) are disproportionately affected by the HIV epidemic, with the proportion of HIV new infections that continue to rise in many regions despite advancements in prevention and treatment. The incidence of HIV among MSM has increased significantly, from 2.5% of newly reported cases in 2006 to 26% in 2014 (1). Data from the Chinese Center for Disease Control and Prevention also indicate an increasing trend in HIV prevalence among MSM in China, rising from 6.9% in 2019 to 8.0% in 2020 (2). It was reported that homosexual transmission accounted for 23.3% of HIV infections in China in 2020 (3). The global prevalence of HIV among MSM highlights the need for a deeper understanding of the factors that contribute to this vulnerability.

The gut microbiome has emerged as a critical player in the interaction between host health and disease, particularly in the context of HIV infection (4). On one hand, HIV infection and viral replication induce severe disruption of gut-associated lymphoid tissue (GALT), which can lead to alterations in gut microbial diversity and species richness (5–7). On the other hand, the role of gut microbiome in HIV susceptibility is multifaceted. It influences the mucosal immune system, which is the first line of defense against pathogens, and can modulate systemic immunity, thereby affecting the host’s response to infections (8). Studies have shown that the gut microbiome composition in MSM differs significantly from that of the general population (9). In addition, one prospective study has revealed that changes in the gut microbiome of MSM prior to HIV acquirement were associated with increased HIV-1 susceptibility and the progression of AIDS (10). Bioinformatics analysis showed that gut microbiome dysbiosis in MSM impacts several immune-related pathways, including the IL-17 signaling pathway and Th17 cell differentiation (11). Moreover, target genes associated with the MSM gut microbiome were found to be highly expressed in monocytes and lymphocytes, indicating their potential regulatory role in immune cells (11). Dysbiosis of the gut microbiome has been linked to increased local inflammation and immune activation, creating a favorable environment for HIV infection (12). Additionally, the gut microbiome’s metabolic functions are thought to affect the availability of nutrients and the production of metabolites, which in turn can influence HIV replication and immune cell function (13).

The concept of enterotypes was first introduced in 2011, which has been used to classify and understand the functional roles of gut microbiome (14). Enterotype of the host remains relatively stable and is rarely affected by gender, age, and body mass index (14). Enterotype research has expanded our understanding of microbial diversity and its implications for health, with enterotypes being defined by the predominance of specific bacterial genera including *Bacteroides, Prevotella,* an*d Ruminococcus* (14). There are differences in the functions and species compositions of different gut types, and different gut types have different digestive functions and preferences (15). They exhibit varying digestive functions and preferences, often associated with dietary habits (16). Individuals with different dietary patterns typically possess distinct enterotypes, and shifts in enterotype composition are frequently associated with various intestinal, metabolic, and immune disorders (17–19). For example, the diversity and composition of the gut microbiome in COPD patients were significantly altered, with the gut enterotype dominated by *Prevotella* (20). In contrast, *Bacteroides*-2 and *Ruminococcus* are more prevalent in patients with type 2 diabetes. However, the enterotypes of gut microbiome in MSM are unclear. It is also unclear whether sexual behaviors impact enterotypes in MSM (21).

The present study aims to explore the gut enterotype characteristics of HIV-negative MSM. Through multivariate logistic regression analysis, we also investigate the associations between gut enterotypes and high-risk sexual behaviors. This research could provide valuable insights into the modifiable factors influencing gut microbiome composition and contribute to the development of targeted interventions for HIV prevention in MSM populations.

## MATERIALS AND METHODS

### Study participants

HIV-negative men who have sex with men were recruited in this study. Recruitment and sample collection were conducted between Oct 2023 and Nov 2023. All participants shall engage in anal intercourse with men within 6 months before the recruitment. Sexual behaviors were self-reported. Volunteers were excluded when they met one of the following criteria: (i) under 18 years of age, (ii) vegetarian, (iii) being infected with HIV, HBV, or HCV, (iv) being diagnosed with any of the heavy gastrointestinal diseases including ulcers, cancers, and other inflammatory diseases, (v) any gastrointestinal operation history, (vi) being diagnosed with any of the metabolic diseases including diabetes, hypertension, and hyperlipemia, and (vii) history of any antibiotic use 3 months before recruitment.

### Fecal sample collection

Fecal samples were collected by the participants with a sterile scoop and stored in a fecal DNA storage tube filled with a preservative solution at room temperature (CWBio, CW2654M). The preservative solution allows for the storage of host and microbial DNA in fecal samples at room temperature for at least 60 days, with long-term stability achievable at −20°C and −80°C. Additionally, it effectively prevents shifts in microbial composition under room-temperature storage conditions. Fecal samples were delivered and stored at −80°C within 24 h after the visit, awaiting DNA extraction for the 16S rRNA gene sequencing.

### Fecal DNA extraction and 16s rRNA gene sequencing

Total fecal DNA was extracted as follows. A 200 mg fecal sample was transferred to a centrifuge tube containing grinding beads, followed by the addition of 1 mL buffer ATL/PVP-10. The sample was homogenized using a grinding machine (Automatic sample rapid grinder, JXFSTPRP-48, Shanghai Jingxin, China) and incubated at 65°C for 20 min. After incubation, the sample was centrifuged at 14,000 g for 5 min, and the supernatant was transferred to a new tube containing 0.6 µL buffer PCI, then vortexed thoroughly for 15 s. The mixture was centrifuged at 18,213 × *g* for 10 min, and the supernatant was transferred to a deep-well plate containing 600 µL magnetic bead binding solution, 20 µL proteinase K, 5 µL RNase, and 100 µL elution buffer. The deep-well plate was placed in the KingFisher system, and DNA extraction was performed by DNA extraction kit according to the manufacturer’s introduction (MagPure Stool DNA KF Kit B, MD5115-02B, MAGEN, China). Upon completion, the extracted DNA was transferred to a 1.5 mL centrifuge tube for further processing.

Library preparation was performed using 2 × Phanta Max Master Mix (VAZYME, China), and the V3-V4 variable regions of 16S rDNA of bacteria were amplified using degenerate forward (F) and reverse (R) primers. Specifically, the V3-4 regions of the 16S rRNA gene were amplified with the following primers: forward (FWD): ACTCCTACGGGAGGCAGCAG and reverse (REV): GGACTACHVGGGTWTCTAAT. PCR enrichment was conducted in a 50 µL reaction system containing 30 ng template DNA and fusion PCR primers, using the following thermal cycling conditions: 95°C for 3 min, followed by 30 cycles of 95°C for 15 s, 56°C for 15 s, and 72°C for 45 s, with a final extension at 72°C for 5 min. PCR products were purified using DNA magnetic beads (BGI, LB00V60) to remove primer dimers and non-specific amplification products.

The final double-stranded library was denatured to generate a single-stranded library, which was then subjected to a circularization reaction to form single-stranded circular DNA molecules. Any remaining linear single-stranded DNA was digested to ensure complete circularization. The circularized DNA molecules were amplified using phi29-mediated rolling circle amplification (RCA) to generate DNA nanoballs (DNBs). The DNBs were loaded onto a patterned nanoarray, and paired-end (PE) sequencing was performed using the DNBSEQ-G400 PE300 platform (BGI-Shenzhen, China).

Raw sequencing data were processed to obtain high-quality clean reads. Reads with an average Phred quality score below 20 over a 30 bp sliding window were truncated, and those shorter than 75% of their original length after truncation were removed. Reads contaminated with adapter sequences, ambiguous bases (N bases), or exhibiting low complexity were discarded. Quality control and filtering were performed using iTools Fqtools fqcheck (v0.25), cutadapt (v2.6), and readfq (v1.0). After quality control, paired-end reads were merged into tags, and OTU (operational taxonomic unit) clustering was performed using USEARCH (v7.0.1090) at 97% sequence similarity. The OTU representative sequences were then aligned against the RDP database (Release 11.5) for taxonomic annotation using the RDP classifier (v2.2).

### Alpha diversity analysis

Prior to conducting microbial community diversity analyses, the raw OTU count table was rarefied by random subsampling to equal depth observed in the data set using the “rrarefy” function in the R “vegan” package. Alpha diversity was evaluated using Shannon index, Simpson index, Chao1 index, and Ace index, and the beta diversity was calculated based on Bray-Curtis distances using R package vegan v2.6-4 at the OTU level. Wilcoxon rank-sum test was conducted to compare the differences in alpha diversity among groups. *P* < 0.05 was considered statistically significant.

### Enterotype analysis

The enterotypes clustering was performed as previously described. First, Jensen-Shannon dissimilarity among individuals was calculated based on the genus-level taxonomic relative abundance. Then, partitioning around medoid (PAM) clustering on the distance matrix was used to determine clusters. The optimal cluster number was selected according to the Calinski–Harabasz index (CH index), and the cluster number with the largest CH index was selected.

### Microbial function prediction

PICRUSt2 software (v2.2.0-b) was used for the functional predictions of the microbial communities based on 16S rRNA gene sequences. The microbial function prediction was performed based on the KEGG database.

### Random forest machine learning model

The three-category random forest model was constructed by R package randomForest (v4.7-1.1). The data set was randomly divided into the training set and test set; 70% of the samples were used as the training set, and the remaining 30% as the test set. Over-sampling was conducted to solve the imbalance in the training set. Subsequently, the balanced training set was subjected to the model constructions. An internal 5-fold cross-validation was applied in the training set to select the best hyperparameters. The final model was created with the following hyperparameters: ntree = 200 and mtry = 1. The area under the receiver-operating characteristic curve (AUC) was assessed to evaluate the performance of the model in the test set. Given the three-category nature of our outcome, we employed a one-vs-one strategy to calculate the AUC values for each pairwise comparison. The macro-average AUC was used to represent the overall model performance.

## RESULTS

### Characteristics of enrolled participants

Ninety-five HIV-negative MSM were enrolled in this study. The characteristics of these participants are stated in Table 1. The mean age of the enrolled MSM was 31 years, and their mean body mass index (BMI) was 20.9 (SD: 3.44). Thirty-three MSM reported that they conducted only in insertive anal intercourse, 16 MSM engaged only in receptive anal intercourse, and nearly half of these participants had both insertive anal intercourse and receptive anal intercourse within 6 months. Most participants had <5 male sex partners. Six MSM reported they never used condoms during anal intercourse.

**TABLE 1:** Characteristics of enrolled HIV-negative men who have sex with men

Variable	Proportion (*N* = 95)
Anal sex role	
Insertive only	33 (34.7%)
Versatile	46 (48.4%)
Receptive only	16 (16.8%)
Number of male partners	
≤1	35 (36.8%)
2–5	44 (46.3%)
6–9	3 (3.2%)
≥10	13 (13.7%)
Condom	
Never	6 (6.3%)
Sometimes	18 (18.9%)
Always	71 (74.7%)
Commercial sex with men	
No	89 (93.7%)
Yes	6 (6.3%)
Group sex with men	
No	87 (91.6%)
Yes	8 (8.4%)
Sexually transmitted diseases	
No	88 (92.6%)
Yes	7 (7.4%)
Illicit drug	
No	88 (92.6%)
Yes	7 (7.4%)

### Two enterotype clusters of gut microbiome in HIV-negative MSM were identified

**Fig 1:**
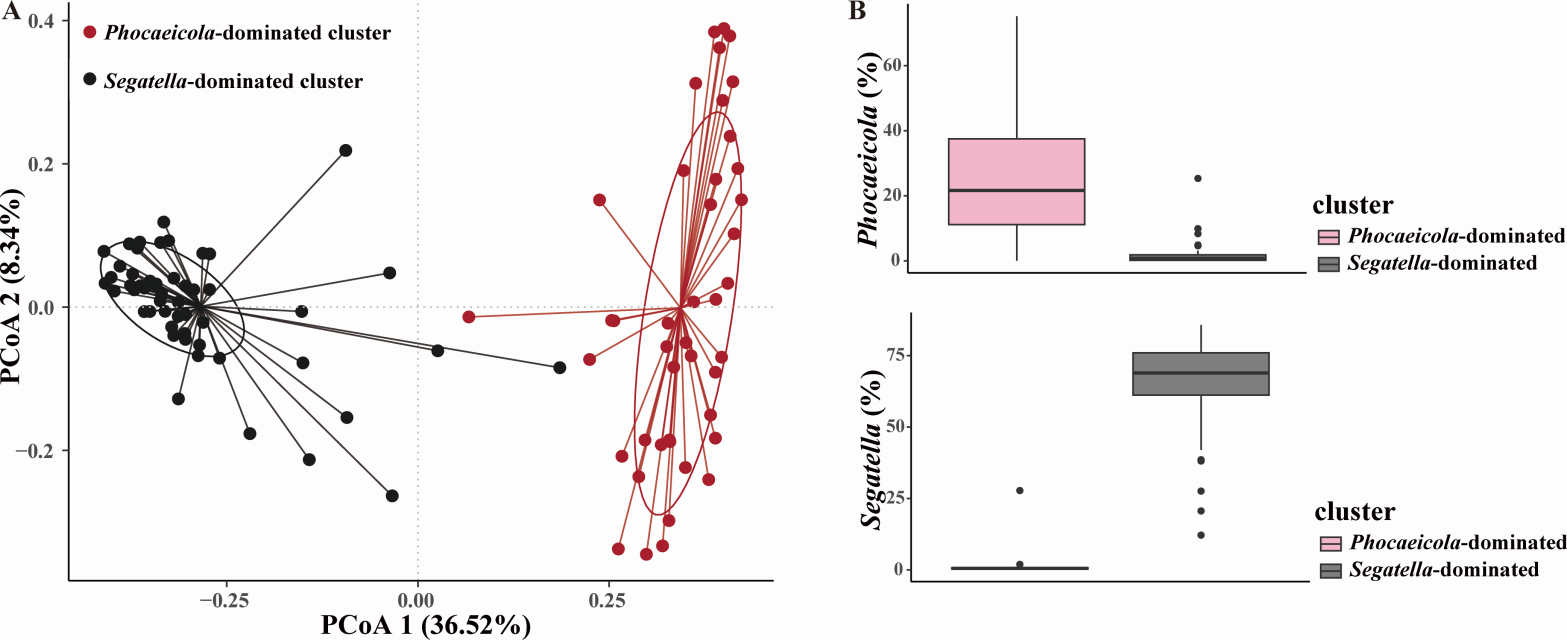
Two gut enterotype clusters were identified in HIV-negative MSM. (**A**) Principal coordinate analysis (PCoA) plot of the two distinctive enterotypes based on fecal microbiome at the genus level. (**B**) The relative abundance of *Phocaeicola* (top) and *Segatella* (down) in *Phocaeicola*-dominated and *Segatella*-dominated clusters.

**Fig 2:**
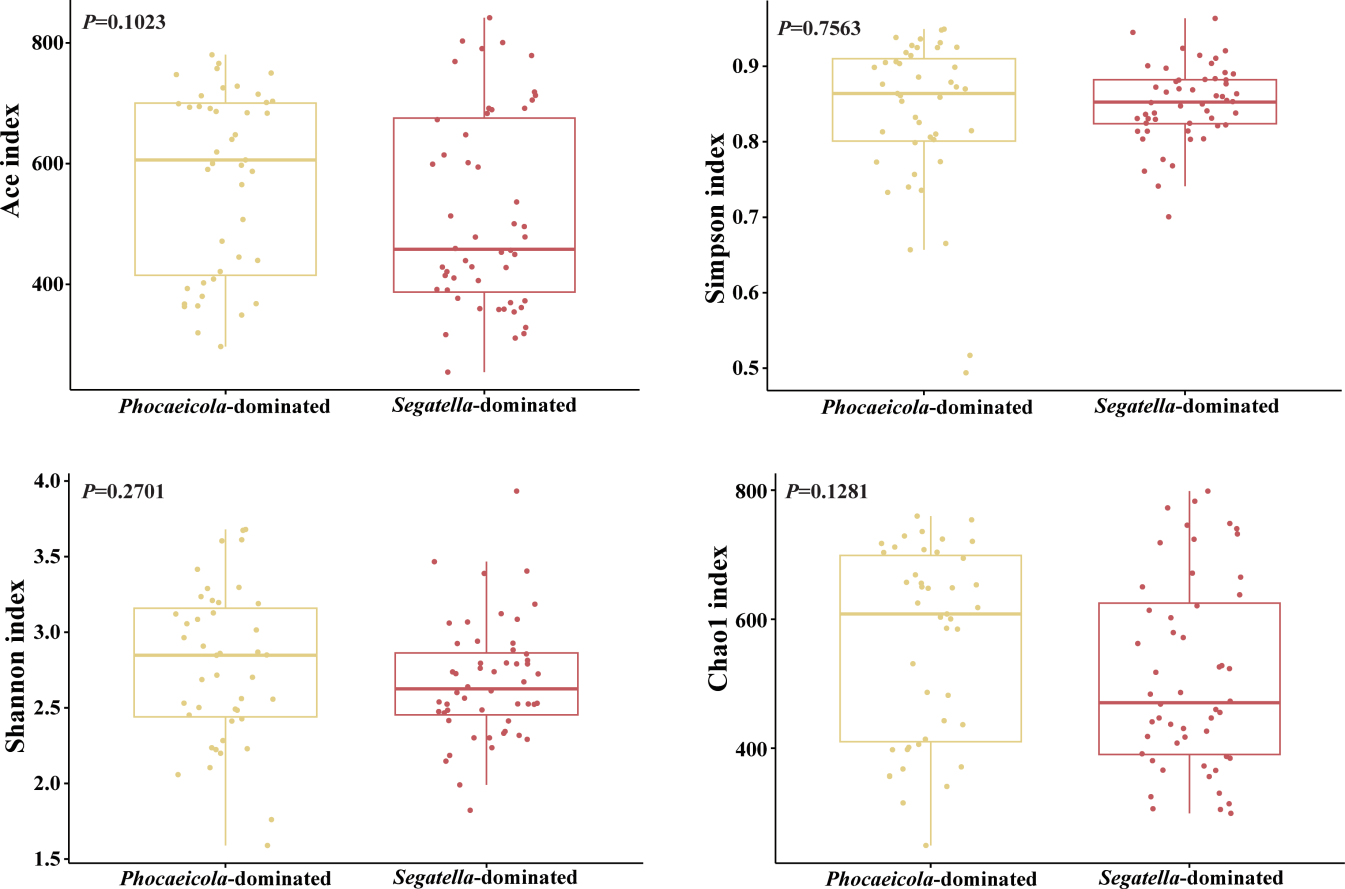
The alpha diversity between Phocaeicola-dominated and Segatella-dominated clusters was comparable.

To assess the overall characteristics of gut microbiome in HIV-negative MSM, we conducted enterotype analysis. Enterotype clusters were defined by partitioning around medoid (PAM) clustering based on Jensen-Shannon distance among samples. As the results showed, two clusters were identified, primarily driven by genera *Phocaeicola* and *Segatella* (Fig. 1). Notably, *Segatella* previously belonged to genus *Prevotella*; thus, this cluster is equal to the prior *Prevotella*-dominated cluster. Forty-three and fifty-two MSM were classified into the *Phocaeicola*-dominated cluster and *Segatella*-dominated cluster, respectively. The demographic characteristics between the two enterotype clusters were similar (Table 2). It is worth noting that the proportion of MSM engaged only in insertive anal intercourse was higher in the *Phocaeicola*-dominated cluster, although the statistic was not significant. Subsequently, we analyzed the microbial alpha diversity in the two clusters. The Shannon index, Ace index, Simpson index, and Chao1 index were not significantly different between the two clusters, which suggested comparable alpha diversity (Fig. 2).

**TABLE 2:** Differences of characteristics between *Phocaeicola*-dominated cluster and *Segatella*-dominated cluster

Characteristic	*Phocaeicola*-dominated cluster (*N* = 43)	*Segatella*-dominated cluster (*N* = 52)	*P*-value
Age			
Mean (SD)	30.0 (7.96)	32.0 (8.81)	0.269
BMI			
Mean (SD)	20.7 (3.87)	21.1 (3.07)	0.591
Anal sex role			
Insertive only	19 (44.2%)	14 (26.9%)	0.056
Versatile	15 (34.9%)	31 (59.6%)	
Receptive only	9 (20.9%)	7 (13.5%)	
Number of male sex partners			
≤1	18 (41.9%)	17 (32.7%)	0.467
2–5	19 (44.2%)	25 (48.1%)	
6–9	0 (0%)	3 (5.8%)	
≥10	6 (14.0%)	7 (13.5%)	
Condom			
Never	4 (9.3%)	2 (3.8%)	0.329
Sometimes	10 (23.3%)	8 (15.4%)	
Always	29 (67.4%)	42 (80.8%)	
Commercial sex with men			
No	40 (93.0%)	49 (94.2%)	0.999
Yes	3 (7.0%)	3 (5.8%)	
Group sex with men			
No	40 (93.0%)	47 (90.4%)	0.725
Often	3 (7.0%)	5 (9.6%)	
Sexually transmitted diseases			
No	40 (93.0%)	48 (92.3%)	0.999
Yes	3 (7.0%)	4 (7.7%)	
Illicit drug			
No	41 (95.3%)	47 (90.4%)	0.451
Yes	2 (4.7%)	5 (9.6%)	

### Microbial functions significantly differed between enterotype clusters

**Fig 3:**
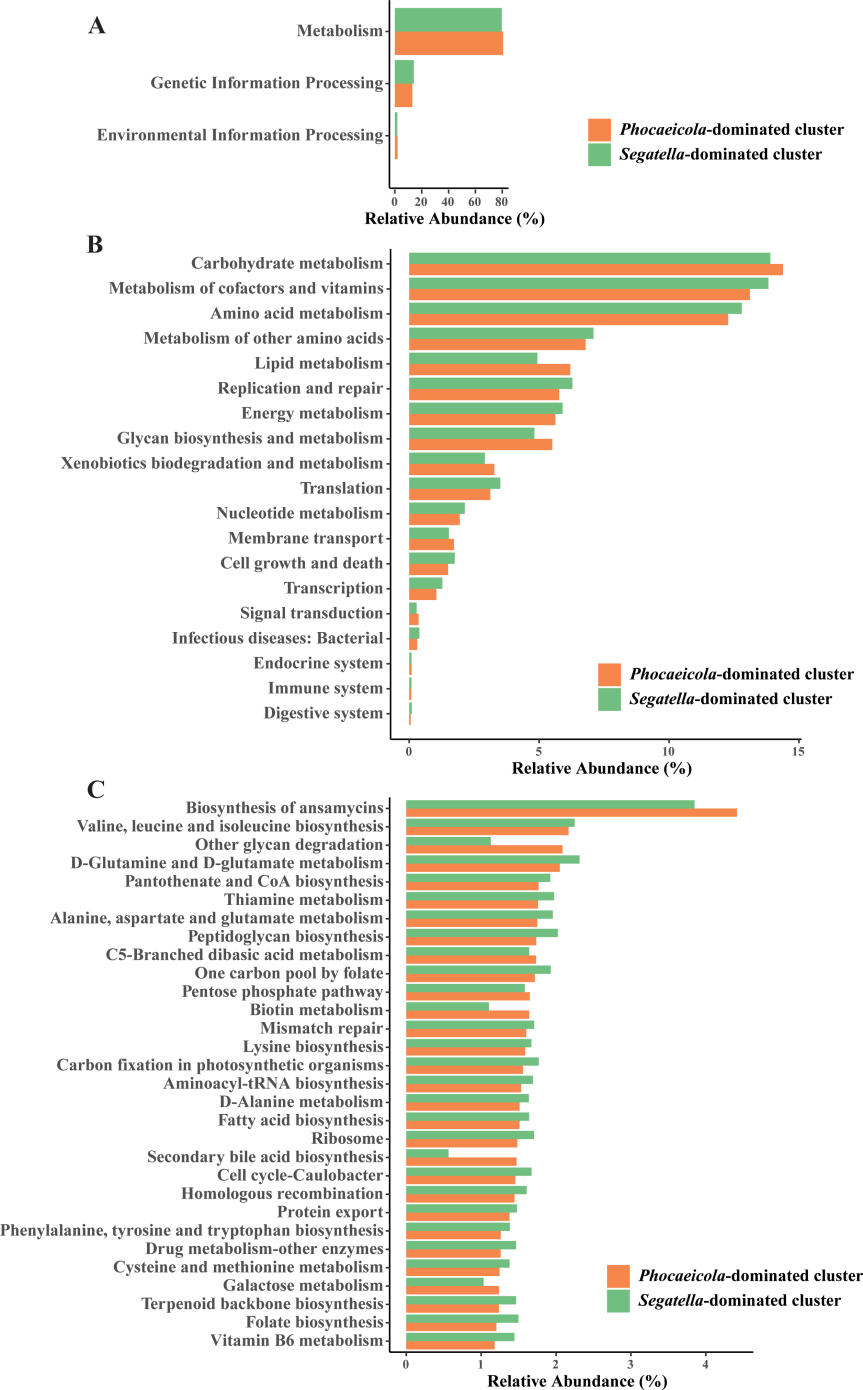
Microbial functions significantly differed between the two enterotype clusters. Predicted KEGG functional pathways at level 1 (**A**), level 2 (**B**), and level 3 (**C**), respectively.

Microbial functions were predicted by PICRUSt2 based on the KEGG database (Fig. 3). At level 1, metabolism was the most abundant function in each enterotype cluster. Compared with the *Phocaeicola*-dominated cluster, the metabolism and environmental information-processing pathways were significantly depleted in the *Segatella*-dominated cluster (*P* < 0.001), whereas the genetic information-processing pathway was increased. At level 2, carbohydrate metabolism, amino acid metabolism, and lipid metabolism were significantly different within the two clusters. Carbohydrate metabolism and lipid metabolism were reduced in the *Segatella*-dominated cluster, and amino acid metabolism was significantly increased. In addition, the pathway related to infectious diseases was also increased in the *Segatella*-dominated cluster. For more detail, we further analyzed the differences in microbial functions at level 3. Among the 176 metabolic pathways, 116 (65.91%) pathways were statistically different between the two enterotype clusters. The top 30 differently enriched pathways included biosynthesis of ansamycins, D−glutamine and D−glutamate metabolism, pantothenate and CoA biosynthesis, fatty acid biosynthesis, and so on. These results showed significant discrepancies in microbial functions between *Phocaeicola*-dominated cluster and *Segatella*-dominated cluster.

### Anal sex roles were significantly associated with enterotypes

**Fig 4:**
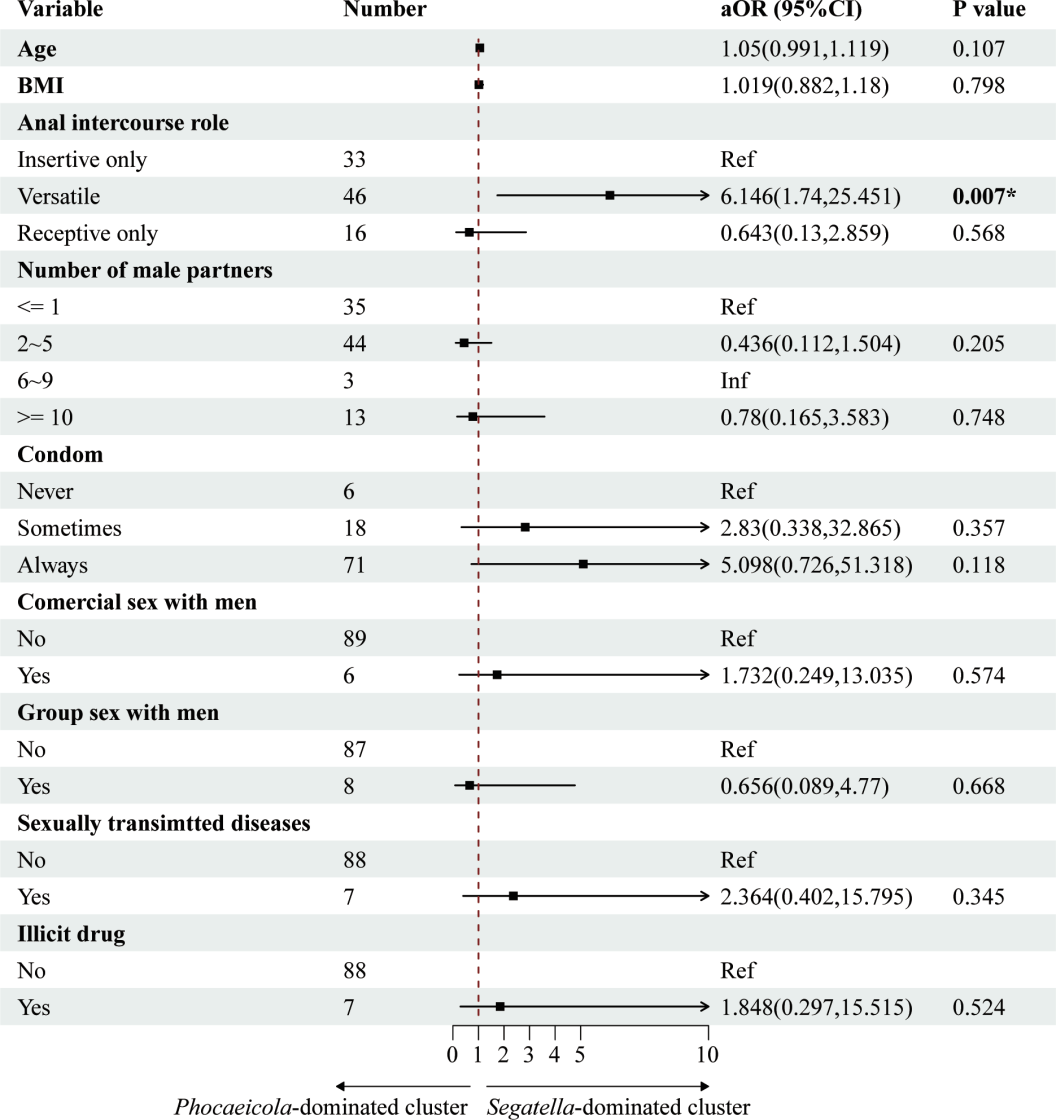
Forest plot showing the results of multivariate logistic regression analysis. Anal sex role significantly affected the gut enterotype in HIV-negative MSM.

The univariate analysis indicated insignificant associations between gut enterotypes and high-risk sexual behaviors. However, the results of univariate analysis could be confounded by other factors. Thus, we conducted a multivariate logistic analysis to evaluate the impacts of sexual behaviors on gut enterotypes (Fig. 4). Importantly, age and body mass index were included in the model as influencing covariates. The results of the multivariate logistic model revealed that the anal sex role was independently associated with enterotype clusters. Compared with MSM who engaged only in insertive anal intercourse, MSM who engaged in both insertive and receptive anal intercourse were concentrated in the *Segatella*-dominated cluster (OR: 6.146, 95% CI: 1.74, 25.451, *P* = 0.007). Although in the subgroup of MSM who engaged only in receptive anal intercourse, the differences in enterotype distribution were not significant, compared with MSM engaged only in insertive anal intercourse, which might result from the limited sample size. We did not observe significant associations between enterotype and age or BMI. Other high-risk sexual behaviors, such as number of male partners, commercial sex with men, and condom use, were not associated with enterotypes in this study (*P* > 0.05).

### Machine learning model revealed nonnegligible associations between the gut microbiome and anal intercourse

**Fig 5:**
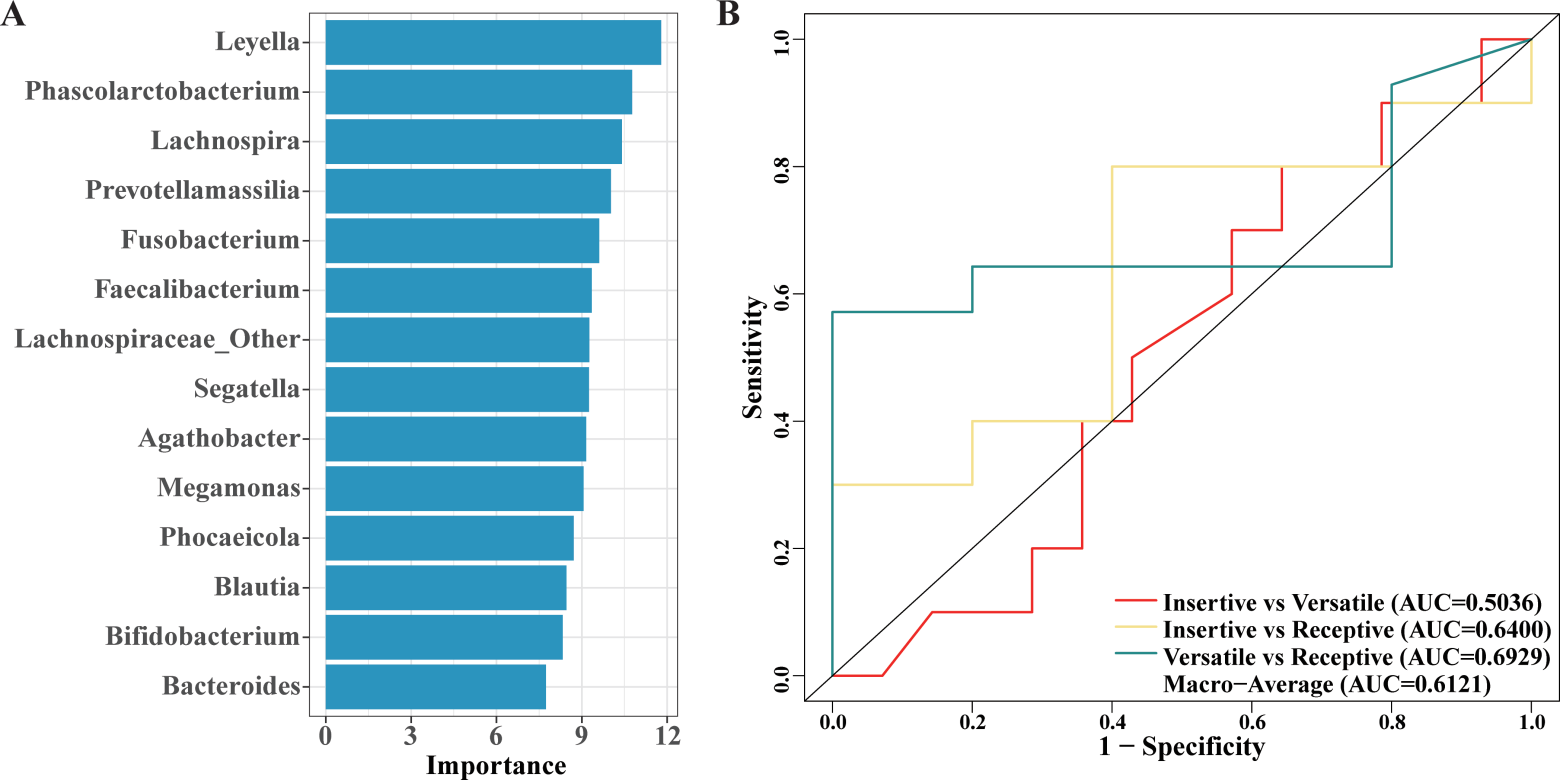
Random forest analysis depicted that the top 10 dominant genera of the two enterotypes could distinguish different sex roles. (**A**) Relative variable importance of the RF model using the permutation-based parameter importance scores. (**B**) Performance assessment of RF models by AUC in the test data set. One-vs-one strategy was used to calculate the AUC values for each pairwise comparison. The macro-average AUC indicated the overall performance of the RF model.

We developed a three-category machine learning model to examine the associations between anal sex roles and the dominant gut microbial taxa in each cluster. The relative importance of each feature in the final model was depicted in Fig. 5A. The one-vs-one strategy was applied for pairwise comparisons among the three categories. The AUC values for each comparison were as follows: Insertive vs. Versatile (AUC = 0.5036), Insertive vs. Receptive (AUC = 0.6400), and Versatile vs. Receptive (AUC = 0.6929). The model demonstrated moderate ability in classifying the different anal sex roles, with a macro-average AUC of 0.6121 across all comparisons (Fig. 5B). The results also indicate that the model exhibited the highest discriminatory ability in distinguishing between versatile and receptive categories, with a moderate ability to differentiate between insertive and receptive categories. The discriminatory ability between insertive and versatile categories was lower. These results further demonstrated the close correlations between anal sex roles and gut microbiome in HIV-negative MSM.

## DISCUSSION

The present study provides a comprehensive analysis of the gut microbiome in HIV-negative MSM, revealing two distinct enterotype clusters that are significantly associated with sexual behavior. Alpha diversity and microbial function in the two clusters were analyzed and compared. Our findings contribute to the growing body of literature that highlights the role of the gut microbiome in HIV susceptibility and underscores the importance of considering sexual behavior as a key factor in microbiome research (10). Our identification of *Phocaeicola* and *Segatella* as the primary drivers of enterotype clustering in HIV-negative MSM is consistent with previous research that has linked these genera to sexual behavior and HIV risk (9). The functional differences between these enterotype clusters, particularly in metabolism, demonstrate significant differences in microbial functional potentials and highlight the distinct characteristics of the two enterotypes. The findings of our study indicate that in *Segatella*-dominated microbiome, there is a reduction in carbohydrate and lipid metabolism, whereas amino acid metabolism is significantly enhanced. The depletion of metabolic pathways in this cluster may create a more conducive environment for HIV infection.

The genus *Segatella* is derived from the original genus *Prevotella* (22). *Prevotella* plays an important role in variable diseases, including inflammation, opportunistic infections, and autoimmune diseases (23). Researchers also reported that a higher abundance of *Prevotella* was associated with increased HPV infection risk and activated NF-KB signaling pathway in women, demonstrating that *Prevotella* could promote virus infection and regulate immune activation (24). In addition, the increased abundance of *Prevotella* populations in the intestinal mucosa is correlated with the activation level of colonic myeloid dendritic cells (mDCs) *in vivo* and can induce colonic mDCs to produce strong proinflammatory cytokines *in vitro*, which may lead to HIV-associated mucosal inflammation and immune activation (25). In our study, we identified a gut enterotype cluster dominated by *Segatella*. Along with previous evidence, our study hinted that this subgroup might be more vulnerable to HIV infection.

The independent association of anal sex role on enterotype clusters is a novel finding. MSM who engage in both insertive and receptive anal sex were more likely to belong to the *Segatella*-dominant cluster. Among MSM, specific sexual behaviors, such as condomless receptive anal intercourse (CRAI), are associated with distinct changes in the gut microbiome (26). Particularly, receptive anal intercourse contributed to an increase in members of the *Prevotella* genus and a decrease in beneficial bacteria such as *Bacteroides* spp (8, 27). These alterations may influence the microbiome composition by increasing the exchange and infection rates of pathogens. However, these studies were primarily designed for the purpose of investigating the effects of HIV status or sexual orientation (MSM or non-MSM) on gut microbiome. Our study focused on the gut microbial community of HIV-negative MSM, which could eliminate the influence of HIV status on gut microbiome. Moreover, the present study primarily explored the association between gut enterotypes and sexual behavior, which is an important gut microbiome characteristic that has been rarely reported in prior studies. Receptive anal intercourse may be attributed to gut dysbiosis through mucosal injury. The rectal mucosal transcriptome, examined 24 h after CRAI, revealed upregulation of genes related to tissue remodeling, neutrophil function, DNA proliferation, and antigen presentation, highlighting the immunological impact of CRAI (28).

According to the results from the PICRUSt2 analysis, carbohydrate metabolism and lipid metabolism were reduced, whereas amino acid metabolism was significantly increased in the *Segatella*-dominated cluster. Impaired carbohydrate metabolism in MSM may influence HIV infection. Carbohydrates account for half of the molecular mass of the outer envelope glycoprotein gp120 of HIV, which covers a large surface area of the envelope and plays an important protective role in HIV immune evasion (29). Lipids such as PI(4, 5)P_2_ not only play an important role in the assembly and maturation of the HIV virus but may also influence the physical properties and infectivity of the virus (30). The amino acid fragment HBA1 (111-132) isolated from human bone marrow has been shown to reduce the viral yield of laboratory-adapted HIV-1 and primary HIV-1 (31). In terms of level 3 results, MSM belonging to the *Segatella*-dominated group were more enriched in the D-glutamine and D-glutamate metabolism pathway than the *Phocaeicola*-dominated group. Changes in glutamine and glutamate metabolism may play an important role in the pathophysiological process of HIV infection by affecting oxidative stress and neurocognitive function (8). These may explain the higher risk of HIV infection among MSM belonging to the *Segatella*-dominated group.

However, our study also had limitations. First, our sample size is relatively small, consisting of only 95 participants. Previous studies have indicated that the number of sexual partners can influence the alpha diversity of the gut microbiome in the MSM population (8). However, our study did not observe these effects, which may be attributed to the limited sample size we recruited.

To sum up, the present study identified two distinct enterotype clusters in HIV-negative MSM, which might represent different risk profiles for HIV acquisition. In addition, receptive anal intercourse exerted an association with gut enterotype among this population. Our study underscores the need for further exploration into the mechanistic links between gut microbiome, sexual practices, and HIV infection in MSM, with the goal of informing preventive strategies for this vulnerable population. The potential for gut microbiome-based interventions to reduce HIV susceptibility in MSM is a promising area for future research.

## Data Availability

The raw sequencing data of this study have been deposited in the NCBI Sequence Read Archive under accession number PRJNA1191636.

## SUPPLEMENTAL MATERIAL

Supplemental material msphere.00232-25-s0001.docx R code for machine learning.

ASM does not own the copyrights to Supplemental Material that may be linked to, or accessed through, an article. The authors have granted ASM a non-exclusive, world-wide license to publish the Supplemental Material files. Please contact the corresponding author directly for reuse.
