# Supplementary material for: Significant associations between high-risk sexual behaviors and enterotypes of gut microbiome in HIV-negative men who have sex with men
Source: mSphere. 2025 Jun 25;10(7):e00232-25. doi: 10.1128/msphere.00232-25 (PMC12306172; doi:10.1128/msphere.00232-25)
Supplement: Supplemental material — R code for machine learning. [file msphere.00232-25-s0001.docx]

**Supplement 1. R code for machine learning in our study**

##random forest machine learning model for three classes

library(randomForest)

library(pROC)

library(caret)

library(dplyr)

library(UBL)

library(ggplot2)

load("./data/data for machine learning 12.5.Rdata")

##Partition data set

set.seed(112)

sample <- sample.split(data$sex_role, SplitRatio = 0.7)

#index <- sample(nrow(data), nrow(data)*0.7)

#training <- data[index, ]

#testing <- data[-index, ]

training <- subset(data, sample == TRUE)

testing <- subset(data, sample == FALSE)

##deal with the imbalance of class

set.seed(111)

train_over <- SmoteClassif(sex_role ~ .,

dat = training,

C.perc = list(versatile = 2, Receptive =6,

Insertive = 3),

k = 5)

# using the five-fold cross-validation to select the optimal hyperparameters

set.seed(11)

folds <- createFolds(train_over$sex_role, k = 5, returnTrain = FALSE)

param_grid <- expand.grid(

mtry = c(1:14),

ntree = seq(10, 300, 10)

)

#create a matrix to record the results

cv_results <- matrix(0, nrow = nrow(param_grid), ncol = 3)

colnames(cv_results) <- c("mtry", "ntree", "accuracy")

# optimization

set.seed(123)

for (i in 1:nrow(param_grid)) {

current_param <- param_grid[i, ]

cv_results_per_param <- c()

for (fold in folds) {

train_data <- train_over[fold, ]

test_data <- train_over[-fold, ]

rf_model <- randomForest(sex_role ~ ., data = train_data, mtry = current_param$mtry,

ntree = current_param$ntree)

predictions <- predict(rf_model, newdata = test_data)

accuracy <- sum(predictions == test_data$sex_role) / nrow(test_data)

cv_results_per_param <- c(cv_results_per_param, accuracy)

}

mean_accuracy <- mean(cv_results_per_param)

cv_results[i, "mtry"] <- current_param$mtry

cv_results[i, "ntree"] <- current_param$ntree

cv_results[i, "accuracy"] <- mean_accuracy

}

print(cv_results)

best_param <- param_grid[which.max(cv_results[, 3]), ]

print(best_param)##mtry=1, ntree=200

##construct the final model with the optimal hyperparameters

set.seed(123)

rf <- randomForest(sex_role ~ ., data = train_over, mtry = 1, ntree = 200)

##one-vs-one strategy to calculate the AUC values for each pairwise comparison

pred_prob <- predict(rf, testing, type = "prob")

classes <- levels(testing$sex_role)

pairs <- combn(classes, 2, simplify = FALSE)

auc_results <- list()

roc_list <- list()

for (i in 1:length(pairs)) {

class1 <- pairs[[i]][1]

class2 <- pairs[[i]][2]

idx <- testing$sex_role %in% c(class1, class2)

true_labels <- testing$sex_role[idx]

prob_class1 <- pred_prob[idx, class1]

true_binary <- ifelse(true_labels == class1, 1, 0)

roc_list[[i]] <- roc(true_binary, prob_class1)

auc_value <- auc(roc_list[[i]])

key <- paste(class1, "vs", class2)

auc_results[[key]] <- auc_value

}
